# High-Throughput Production of Micrometer Sized Double Emulsions and Microgel Capsules in Parallelized 3D Printed Microfluidic Devices

**DOI:** 10.3390/polym11111887

**Published:** 2019-11-15

**Authors:** Alexander Jans, Jonas Lölsberg, Abdolrahman Omidinia-Anarkoli, Robin Viermann, Martin Möller, Laura De Laporte, Matthias Wessling, Alexander J. C. Kuehne

**Affiliations:** 1 DWI—Leibniz Institute for Interactive Materials, Forckenbeckstraße 50, 52076 Aachen, Germany; jans@dwi.rwth-aachen.de (A.J.); loelsberg@dwi.rwth-aachen.de (J.L.); omidinia@dwi.rwth-aachen.de (A.O.-A.); robin.viermann@rwth-aachen.de (R.V.); moeller@dwi.rwth-aachen.de (M.M.); delaporte@dwi.rwth-aachen.de (L.D.L.); wessling@dwi.rwth-aachen.de (M.W.); 2 AVT—Chemical Process Engineering, RWTH Aachen University, Forckenbeckstraße 51, 52074 Aachen, Germany; 3 OC3—Institute of Organic and Macromolecular Chemistry, Ulm University, Albert-Einstein-Allee 11, 89081 Ulm, Germany

**Keywords:** microfluidics, rapid prototyping, 3D printing, capillary, hollow microgels

## Abstract

Double emulsions are useful geometries as templates for core-shell particles, hollow sphere capsules, and for the production of biomedical delivery vehicles. In microfluidics, two approaches are currently being pursued for the preparation of microfluidic double emulsion devices. The first approach utilizes soft lithography, where many identical double-flow-focusing channel geometries are produced in a hydrophobic silicone matrix. This technique requires selective surface modification of the respective channel sections to facilitate alternating wetting conditions of the channel walls to obtain monodisperse double emulsion droplets. The second technique relies on tapered glass capillaries, which are coaxially aligned, so that double emulsions are produced after flow focusing of two co-flowing streams. This technique does not require surface modification of the capillaries, as only the continuous phase is in contact with the emulsifying orifice; however, these devices cannot be fabricated in a reproducible manner, which results in polydisperse double emulsion droplets, if these capillary devices were to be parallelized. Here, we present 3D printing as a means to generate four identical and parallelized capillary device architectures, which produce monodisperse double emulsions with droplet diameters in the range of 500 µm. We demonstrate high throughput synthesis of W/O/W and O/W/O double emulsions, without the need for time-consuming surface treatment of the 3D printed microfluidic device architecture. Finally, we show that we can apply this device platform to generate hollow sphere microgels.

## 1. Introduction

Droplet-based microfluidics are a versatile tool to generate double emulsions, which is a droplet inside a droplet in a continuous phase [1]. In contrast to bulk emulsification techniques, droplet-based microfluidics produce one droplet after the other, allowing precise tuning of the droplet diameters and the generation of highly monodisperse emulsions and double emulsions [2,3]. Therefore, microfluidic double emulsion technology is a powerful tool to generate vesicles [4,5], core-shell particles [6], and hollow sphere particles and microgels [7] to encapsulate therapeutic agents [8] or cells [9,10]. The most widely applied technology platforms to generate double emulsions are glass capillary devices [11] on the one hand and channel geometries produced by soft-lithography in silicones (PDMS) [12,13] on the other hand. For the preparation of capillary double emulsion devices, two differently tapered glass capillaries are aligned so that their tips face each other. The tapering and alignment processes usually go in hand with mismatches, which in a parallelized device causes polydispersity due to different pressure drops and disturbed flow patterns at edges and corners [14]. By contrast, double emulsion drop makers can easily be parallelized by soft lithography. The inlets and outlets for fluid delivery to and from the microfluidic chip can be connected using wide distribution and collection channels. This idea has brought forth various designs for upscaling the production of double emulsions, namely *tree*- or *ladder*-type channel geometries [15] as well as devices for tandem emulsification [16] and also partially 3D printed junction parts [17]. In glass capillary devices, the fluids wet complete sections of the capillaries and wetting conditions do not change. For example, only the continuous phase is in contact with the emulsifying orifice as well as the inside and outside of the collection capillary. By contrast, the fluids in a device prepared by soft-lithography alternatingly wet the channel walls. This requires modification of the respective surface chemistry (hydrophilicity/hydrophobicity) of the channel section to produce uniform droplet sizes. However, surface modification becomes increasingly difficult with the number of parallelized drop makers. Furthermore, the two-dimensional nature of devices produced by soft lithography entails large footprints and dead volumes because the individual drop makers need to be connected using distribution and collection channels. As a result, both approaches exhibit severe drawbacks, which complicate parallelization and therefore inhibit their wide-spread application in industrial processes.

An ideal geometry for microfluidic parallelization of double emulsion devices needs to combine the benefits of the two contrasting technologies, namely provide precise reproducibility of the channel geometry as in the soft-lithography approach coupled with a three-dimensional flow profile like in capillary microfluidics to prevent alternating wetting conditions along the channel path. However, due to these apparently excluding requirements, such devices have been absent to date, and upscaling of uniform double emulsions remains a formidable challenge.

Previously we have shown parallelization in 3D printed channel geometries for single emulsions [18]. Here we present a microfluidic double emulsion device with a three-dimensional channel geometry produced by 3D rapid prototyping. We combine a tapered (capillary-like) channel geometry for droplet production with a compact channel layout, resulting in a small double emulsion device with minimal footprint and little dead volume. The precise resolution and reproducibility of 3D printing allows us to parallelize multiple double emulsion channels in one device. Our devices do not require any priming for surface modification or compatibilization, which we demonstrate by producing monodisperse W/O/W as well as O/W/O emulsions in the same device. We demonstrate high throughput in a device where we parallelized four drop maker and operate it at high gauge pressures of one bar, allowing us to produce monodisperse double and multiple emulsions with diameters of hundreds of microns at rates of 1.2 L/h.

## 2. Results and Discussion

**Figure 1:**
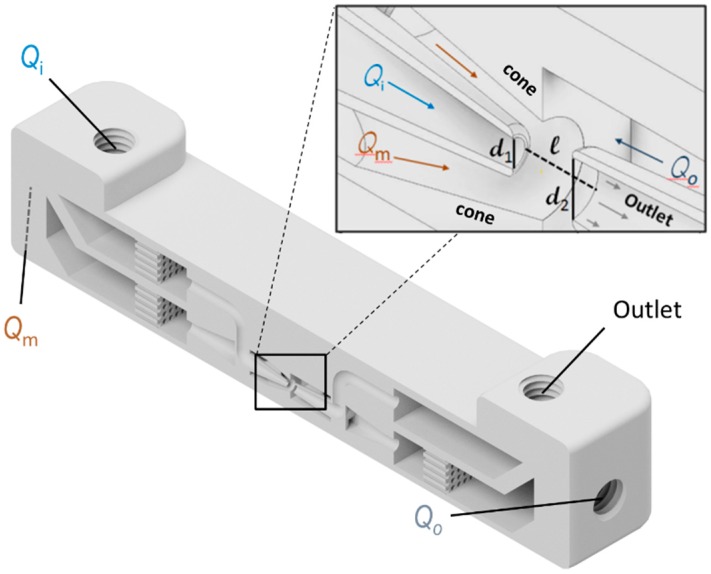
Lateral cut view on a 3D rendering of the parallelized device with one of the four double emulsion drop makers and the respective distribution channels for three inlets and a collection channel. Each one of these wider distribution and collection channels ends in a printed M5 thread to connect the chip with tubing to the outside world. The inset shows a rendered close-up of the droplet generation site inside of the 3D printed microfluidic double emulsion device. *Q*_i_ shows the inlet of the inner phase, *Q*_m_ of the middle phase, *Q*_o_ of the continuous outer phase, and finally the outlet for collecting the double emulsions is indicated.

**Figure 2:**
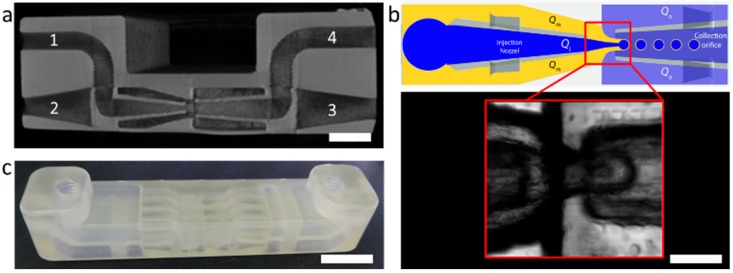
(**a**) µCT scan of the double emulsification element. The acrylate material appears in light gray color and the channel structure in darker contrast. The numbers indicate the in- and outlets: (**1**) inlet fluid for inner droplet, (**2**) inlet for midlle phase (shell droplet), (**3**) inlet continuous phase, (**4**) outlet for sample collection. (**b**) Schematic of the double emulsification process with symbols for driving pressures of the respective inlets. The zoomed inset displays a brighfield microscopy image of the droplet generation zone showing the formation of a double emulsion droplet. (**c**) Photograph of the 4-fold parallelized doulbe emulsion drop-maker device with connection points for push-in fittings. Scale bars represent (**a**) 1.5 mm, (**b**) 450 µm, and (**c**) 5 mm.

To obtain a geometry for optimal double emulsification, we adhere to previously described theory to set the distance and ratio of the capillary orifices [19,20,21]. We are limited by the resolution of our 3D printer (Stratasys, Rechovot, Israel, Object Eden 260V) with nominal resolution limits of 32 µm in x and y and 16 µm in z-direction. With these parameters in mind we iteratively optimize the device geometry with regards to minimal inner diameter of the capillaries, minimal capillary wall thickness, as well as distance between the coaxial inner phase capillary and the collection capillary. This generative optimization leads to diameter *d*_1_ = 450 µm for the spherical orifice of the inner phase capillary, a diameter of *d*_2_ = 900 µm for the collection capillary, and a distance of *l* = 1000 µm between them (see Figure 1). In contrast to classical capillary devices, where the geometry is set by the shape of the two opposing tapers and a rectangular encasing capillary, 3D printing allows us to modify the droplet generation site further. We found that geometrical focusing (as opposed to fluid focusing in the glass capillary device) of the middle phase enables greater stability for droplet generation over a wider interval of fluid flow rates. This can be achieved by incorporating a cone around the tapered capillary of the inner fluid (see inset in Figure 1), thereby locally stabilizing the phase boundary. The entire drop maker device is shown in Figure 1. Here it becomes clear how the intricate taper geometry is supported by the surrounding channel geometry, which allows a combination of three fluid inlets and one collection outlet on a small base area (see Figure 2a).

For parallelization, the central double emulsification element is connected with distribution channels much wider than the channels leading to the droplet generation site. This allows for minimal pressure drop along the element inlets and uniform droplet formation in all connected double emulsion drop-makers (see Supplementary Information for STL-file). We print the device in acrylic monomer, which is polymerized using an admixed photoinitiator (VeroClear^TM^ RGD810 by Stratasys, Rechovot, Israel). Upon irradiation, a thermoset is produced with good chemical stability towards aqueous and most organic solvents. To remove unreacted resin in the channels, we flush the device with aqueous NaOH solution (see methods for parameters). Subsequently, the cleaned device is subjected to flood exposure of 302 nm for 1 h to activate the residual photoinitiator and hard-cure the device, to give it additional mechanical integrity.

To evaluate the quality of the print and resist removal protocol, we performed X-ray microtomography (µCT) to reveal the internal structure and accessibility of all channels in our device. The µCT image reveals an open channel geometry where the acrylate material appears as gray contrast (see Figure 2a).

**Figure 3:**
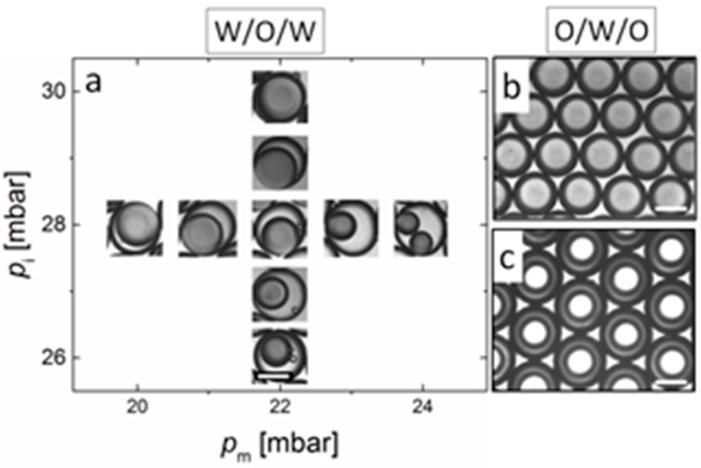
(**a**) Phase diagram for W/O/W emulsions with varying pressures for inner *p*_i_ and middle phase *p*_m_. *p*_o_ was kept constant at 200 mbar. (**b**) monodisperse emulsions of O/W/O with large inner droplet obtained at *p*_i_ =23 mbar, *p*_m_ = 19 mbar and (**c**) monodisperse O/W/O emulsion with small inner droplet obtained at *p*_i_ = 24 mbar, *p*_m_ = 27 mbar. *p*_o_ in (**a**–**c**) was set to 200 mbar. Scale bars represent 500 µm.

To showcase the applicability of the device, we prepared a parallelized device with 4 double emulsion drop makers (Figure 2c). We produced W/O/W double emulsions by injecting an inner phase of 2 wt% Tween 80 in water with a blue dye for better contrast, a middle phase of hexane and paraffin (1:1) with 2 wt% Span 80 and again aqueous 2 wt% Tween 80 solution as the continuous phase (see Figure 2b). To display that we can vary the core and shell thickness by adjusting the flow rates, we tuned the driving pressure *p*_i_ for the inner phase between 26 and 30 mbar and for the middle phase *p*_m_ between 20 and 24 mbar, while keeping the pressure of the continuous phase constant at *p*_o_ = 200 mbar. While the generation of larger and smaller overall droplet diameters is possible in our 3D printed device through variation of *p*_o_, stable operation is merely possible for droplet diameters of the order of the orifice diameter [19]. By varying the driving pressures *p*_i_ and *p*_m_, the ratio of the inner droplet to shell size can be tuned while the overall size of the double emulsion droplet remains around 500 µm, predetermined by the orifice diameter of the collection channel (Figure 2b inset). This way, the shell thickness can be varied from ~ 20 to 250 µm by either increasing the driving pressure of the middle oil fluid or decreasing the pressure of the inner aqueous phase (Figure 3). For higher middle phase pressures, multiple aqueous droplets can be produced inside the oil droplet (see Figure 3a). For constant pressure operation, we obtain monodisperse double emulsions, in which the outer diameter and inner droplet are uniform in their diameters with relative standard deviations consistently <5%.

To delineate the robustness of the device geometry and prove that surface modification is not necessary irrespective of the emulsified phases, we prepare O/W/O double emulsions using the above described surfactant concentrations in the same device after flushing with isopropanol and drying. Here we stain the middle aqueous phase with a blue dye. We immediately obtain monodisperse double emulsion droplets with tunable shell thicknesses in a similar pressure regime as for the W/O/W system, as shown in Figure 3b,c.

**Figure 4:**
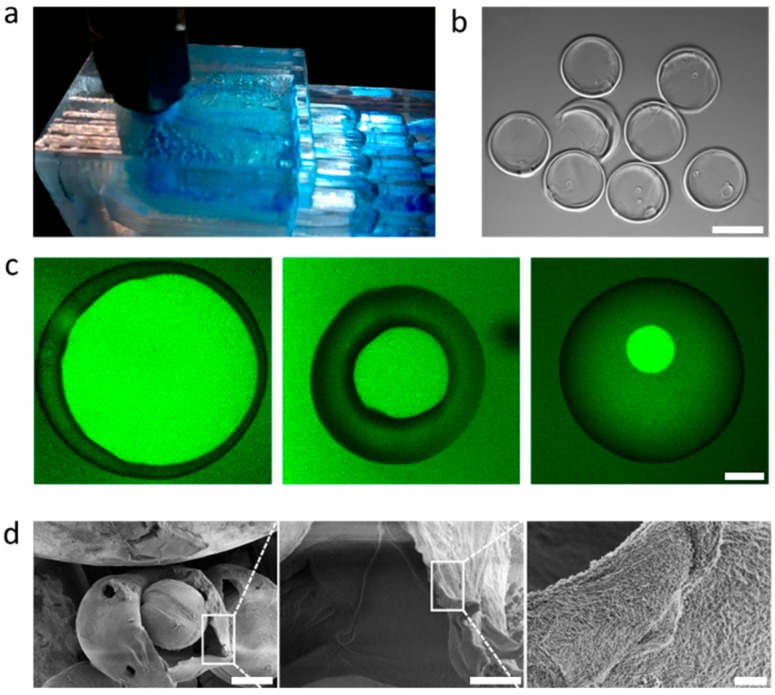
(**a**) Photograph of the outlet and collection channel of a four-fold parallelized device. (**b**) hollow sPEG based microgels of ~ 500 µm in diameter after drying. (**c**) Confocal scanning laser microscope image of hollow microgels labeled with FITC- functionalized dextran. The fluorescent dextran was allowed to diffuse into the polymer network to highlight the inner cavities of different diameters. The microgels were produced at applied pressures of (**left**) *p*_i_ =23 mbar, *p*_m_ = 19 mbar, (**middle**) *p*_i_ =24 mbar, *p*_m_ = 27 mbar, (right) at *p*_i_ =23 mbar, *p*_m_ = 28 mbar. *p*_o_ was set to 200 mbar. (**d**) Cryo-SEM images shows the hollow character with frozen water core. Close up reveals the homogenous polymer network of the capsules. Scale bars represents (**b**) 500 µm, (**c**) 100 µm, (**d**) 150 µm, 25 µm, and 5 µm (**left** to **right**).

These O/W/O emulsion droplets represent ideal reaction templates to produce biocompatible capsules. We admix 10 wt% of an acrylate terminated six-armed poly(ethylene-*co*-propylene glycol) (sPEG *M*_n_ = 18 kDa) and a water-soluble photoinitiator to the aqueous shell phase. The produced double emulsion (O/W/O) with star-PEG in the aqueous phase was exposed to λ = 365 nm to initiate polymerization and produce a water-swollen microgel network. This polymerization was performed using a UV LED illuminating the tubing, which led the double emulsion away from the microfluidic chip. This semi-off-chip polymerization prevents potential stray light and detrimental activation of the photoinitiator inside of the microfluidic device, which could lead to clogging. The collected sample was purified by repeated centrifugation and redispersion using isopropanol, followed by a final dispersion step in water. The production rate for these hollow sphere microgels can be as high as 1.2 L·h^−1^, yielding about 10^7^ of monodisperse microgel capsules per hour (see Figure 4a and Video S1 in Supplementary Information). The microgel capsules have diameters of around 500 µm and represent the largest hollow microgels prepared and reported to date [22,23]. In fact, the microgels are so large, that the specimens with very thin shells collapse upon drying and lead to donut-shaped geometries (see Figure 4b). Interestingly, when swollen these microgel capsules remain mechanically stable independent of the shell thickness, as shown by confocal microscopy where we image a slice through the equator of the microgel capsule (Figure 4c). For imaging, we infiltrated the hollow microgels with fluorescein functionalized dextran (10 kDa) to display that the microgel shell is porous with an open network structure. The dextran diffuses through the network to stain the compartment in the core. Due to the well-known immiscibility of dextran in PEG, we observed the sPEG microgel shell in darker contrast against the fluorescent shell and aqueous background (Figure 4c).

We performed cryo-SEM analysis to reveal homogenous network distribution of the crosslinked sPEG shell and the hollow character of our microgel capsules. During sample preparation in vacuum, some of the microgel capsules burst revealing frozen water droplets in the core of the capsules, which can be clearly distinguished from the collapsed microgel shell (Figure 4d). Zooming in on the microgel shell reveals its homogeneous porosity. Such hollow microgel capsules could find application in delivery vehicles, for example for long term release or encapsulation of cells [7,24,25,26].

## 3. Conclusions

We here extend the platforms of 3D printed parallelized microfluidic devices for the preparation of double emulsions, multi-phase materials, and microgel capsules. We combined the benefits of lithographic patterning with the beneficial wetting characteristics of capillary geometries by utilizing the full 3-dimensional freedom available from 3D printing. Foreseeable improvement of resolution in light-based 3D printing will enable reduction of the feature size and therefore allow production of smaller droplet sizes. The combination of a small footprint, low dead volume, as well as the high reproducibility and excellent solvent resistance of our 3D printed devices outperforms silicone and glass-capillary devices. In the future, our parallelization approach for double emulsion microfluidics presented here could be easily extended and translated to other fluids and device geometries for the generation of novel materials and particle geometries for new applications.

## 4. Materials and Methods

### 4.1. Materials

Paraffin oil, Hexane, Isopropanol, TWEEN 80, SPAN 80, Irgacure 2959 and FITC-Dextran (10 kDa) were purchased from Sigma-Aldrich (Munich, Germany). Tubing was ordered from Smith Medical Inc. (Minneapolis, MN, USA). Connection needles (25G 5/8″) were obtained from Sigma-Aldrich (Munich, Germany).

### 4.2. Methods

#### 4.2.1. Device Preparation

Similar to a protocol described previously [27], the 3D printed microfluidic droplet maker was fabricated using polyjet 3D printing (Objet Eden 260V Stratasys, Rechovot, Israel). The device was printed layer by layer with a transparent photopolymer (VeroClear RGD810, Stratasys, Rechovot, Israel). During printing, the internal fluid channels were supported using Stratasys, SUP705, which we later removed using a high-pressure washer (RK Top 5, Krumm-tec, Endingen am Kaiserstuhl, Germany). Any remaining material was subsequently dissolved in 1 mol·L^−1^ sodium hydroxide for approximately 12 h to remove unreacted resin and supporting structures (produced from PolyJet SUP705, Stratasys, Rechovot, Israel). This was achieved by flushing the device. We connected the device using the printed threading for push-in fittings (Riegler, Bad Urach, Germany) and insert tubing to connect to syringe pumps and flush the device from the inlet of the inner phase and the outlet side. The dissolved resin was washed out through the inlets of the middle and continuous phases.

#### 4.2.2. Emulsification W/O/W—O/W/O

The hydrophobic phase for the emulsification process consisted of paraffin oil and hexane in a ratio of 1:1. To stabilize the generated droplets, 2 wt% of the surfactant SPAN 80 was added. The aqueous phase was prepared by mixing 2 wt% of TWEEN 80 to water (MilliQ). For better visualization, a few drops of blue ink were added.

To generate hollow microgel capsules, 10 wt% of a six arm star-shaped (PE-*stat*-PP) acrylate terminated precursor was dissolved in the aqueous phase. A photoinitiator (Irgacure 2959, 1 wt%) was added to the mixture and stirred for 30 min in the dark to prevent unintended polymerization. The emulsified double emulsion (O/W/O) was polymerized off chip, inside the tubing that was leading away from the outlet, by irradiation with an LED source (λ_max_ = 365 nm). The hollow microgels templates were exposed for a minimum time of 180 s and collected in a glass vial. For purification purposes, colloids were transferred step wise from non-polar solvent to an aqueous media (hexane, 2-propanol, water).

#### 4.2.3. FITC-Dextran Loading of Hollow Microgels

For loading and incubation, a 1 mg/mL FITC-Dextran (10 kDa) was prepared and added to the microgel suspension, which was shaken for 24 h in the dark to facilitate full penetration of the microgel network.

#### 4.2.4. Confocal Laser Scanning Microscopy (CLSM)

Confocal laser scanning microscopy was performed on a Leica TCS SP8. Incubated microgels were dispersed three times in fresh water to remove any excess of FITC-Dextran. A droplet of the freshly dispersed FITC-Dextran loaded microgel capsules was placed on a 22 × 50 mm glass slide. FITC-dextran was excited with an argon laser at λ = 488 nm and the resulting emission was detected at λ = 510–550 nm using Leica HyD detectors.

## Supplementary Materials

The following are available online at https://www.mdpi.com/2073-4360/11/11/1887/s1, STL-file of the parallelized device and Video S1: parallelized double emulsion production.
